# Toward Composite Pain Biomarkers of Neuropathic Pain—Focus on Peripheral Neuropathic Pain

**DOI:** 10.3389/fpain.2022.869215

**Published:** 2022-05-11

**Authors:** Monica M. Diaz, Jacob Caylor, Irina Strigo, Imanuel Lerman, Brook Henry, Eduardo Lopez, Mark S. Wallace, Ronald J. Ellis, Alan N. Simmons, John R. Keltner

**Affiliations:** ^1^Department of Neurology, University of North Carolina at Chapel Hill School of Medicine, Chapel Hill, NC, United States; ^2^Department of Anesthesiology, University of California, San Diego, San Diego, CA, United States; ^3^Department of Psychiatry, San Francisco Weill Institute for Neurosciences, University of California, San Francisco, San Francisco, CA, United States; ^4^Department of Psychiatry, University of California, San Diego, San Diego, CA, United States; ^5^Department of Neurosciences, University of California, San Diego, San Diego, CA, United States; ^6^Department of Psychiatry, San Diego & Center of Excellence in Stress and Mental Health, Veteran Affairs Health Care System, University of California, San Diego, San Diego, CA, United States; ^7^Department of Psychiatry, San Diego & San Diego VA Medical Center, University of California, San Diego, San Diego, CA, United States

**Keywords:** pain, biomarker, neuropathic, endocannabinoid, inflammation, opiate

## Abstract

Chronic pain affects ~10–20% of the U.S. population with an estimated annual cost of $600 billion, the most significant economic cost of any disease to-date. Neuropathic pain is a type of chronic pain that is particularly difficult to manage and leads to significant disability and poor quality of life. Pain biomarkers offer the possibility to develop objective pain-related indicators that may help diagnose, treat, and improve the understanding of neuropathic pain pathophysiology. We review neuropathic pain mechanisms related to opiates, inflammation, and endocannabinoids with the objective of identifying composite biomarkers of neuropathic pain. In the literature, pain biomarkers typically are divided into physiological non-imaging pain biomarkers and brain imaging pain biomarkers. We review both types of biomarker types with the goal of identifying composite pain biomarkers that may improve recognition and treatment of neuropathic pain.

## Introduction

Pain is a protective evolutionary function that involves “unpleasant sensory and emotional experiences associated with, or resembling that associated with, actual or potential tissue damage” (International Association for Study of Pain). Acute pain is an adaptive and essential survival behavior. Chronic pain is a pathological condition that poses a significant clinical, economic and social burden (1, 2). Chronic pain is the most common clinical complaint in the United States affecting ~10–20% of the U.S. population with an estimated annual cost of $600 billion, the most significant economic cost of any disease to-date (2–6).

Neuropathic pain is defined as pain that is “initiated or caused by a primary lesion or dysfunction in the nervous system” (7). Neuropathic pain can be divided into either having peripheral origin or central origin and can be further divided into acute or chronic pain, the latter defined as pain lasting for longer than 3 months (7).

Pain is a subjective sensory experience that cannot be directly measured nor quantified. Although pain is subjective and influenced by many physiological and psychological factors, measuring biomarkers of neuropathic pain provides an opportunity to identify objective markers of peripheral nerve damage and other pathology contributing to neuropathic pain. If used in combination, biomarkers related to pain mechanisms (including opiate, inflammation, and endocannabinoid mechanisms) offer the possibility to develop objective pain-related indicators that may improve diagnosis, treatment, and understanding of pain pathophysiology (8–11). The pursuit of pain biomarkers has followed two largely separate general directions: physiological vs. brain neuroimaging. Physiological pain biomarkers research has followed multiple lines of investigation including genetic, vesicular micro-RNA, metabolic/molecular, and stress markers. Neuroimaging biomarker research in neuropathic pain research was initially motivated by research into brain areas activated by painful stimuli and that vary with pain severity (10, 12–14). Brain activity that occurs in response to pain can also be observed in the absence of pain, which has led to conflicting evidence regarding brain activity related to pain. Thus, some researchers are developing biomarkers based on the mechanisms underlying pain and pain perception and biomarkers that may predict response to medication and pain treatments allowing for prediction of personalized treatment responses (10, 15, 16).

Toward the goal of identifying composite biomarkers for investigating neuropathic pain mechanisms and improving diagnosis and treatment response, we present a review of non-imaging and imaging pain biomarkers related to various neuropathic pain mechanisms, including opiate, inflammation, endocannabinoid mechanisms. In this review, we review mechanisms for neuropathic pain in general, but we focus on pain biomarkers for different types of peripheral neuropathies. Although various reviews of pain biomarkers exist, we focus on creating composite biomarkers through machine learning approaches that can most accurately identify people with neuropathic pain.

## Opioid Pain Biomarkers

### Opioid Mechanisms

Endogenous opioids are necessary for the expression of pain relief (17) and pain-induced aversion (18, 19). Blocking opioidergic transmission reduces dopamine release in the nucleus accumbens that accompanies pain relief (20). The endogenous opioid system consists of four opioid peptide families: β-endorphin, enkephalins, dynorphins, and nociceptin/orphanin and 4 families of receptors: mu, delta, kappa, and nociceptin (21, 22).

Opioid receptors are expressed by central and peripheral neurons, by neuroendocrine (pituitary, adrenals), immune, and ectodermal cells (23–25). All opioid receptor types mediate analgesia but have differing side effects, mostly due to their variable regional expression and functional activity in different parts of central and peripheral organ systems. Endogenous opioids are particularly concentrated in circuits involved in pain modulation (26).

### Opioid Pain Biomarkers

Beta-endorphin levels in the CSF, blood and saliva have been investigated as possible pain biomarkers. Plasma Beta-endorphin has been used to investigate age responses to experimental pain (27). Patients with chronic neuropathic pain due to trauma or surgery have been shown to have lower levels of Beta-endorphin in the CSF (28). Plasma and CSF Beta-endorphin have been investigated in patients with trigeminal neuralgia (29). Interestingly, Beta-endorphin in peripheral blood was related to levels in CSF; furthermore, the levels of Beta-endorphin were inversely correlated with the severity of pain symptoms (29). While chronic low back pain typically involves non-neuropathic pain mechanisms, it is interesting that plasma Beta-endorphin levels have been shown to be a promising biomarker for chronic back pain (30). In other non-neuropathic pain conditions, mu opioid receptors expressed on immune B cells was found to be a biomarker for chronic pain in fibromyalgia and osteoarthritis. In this study, the percentage of mu opioid receptors positive B cells was statistically lower in patients with moderate to severe pain than in pain-free subjects or mild pain subjects (31). In a heterogenous group of patients with pain, a composite biomarker was identified that uses emergent properties in genetics to separate patients with pain requiring extremely high opioid doses from controls (32). Negative studies for opiate mechanism pain biomarkers have shown that salivary Beta-endorphin is not a biomarker for neuropathic chronic pain propensity (33).

Functional brain imaging performed on patients with non-neuropathic primary dysmenorrhea with mu-opioid receptor A118G polymorphism has been used to investigate pain sensitivity and opioid-analgesic treatment related to function in the descending pain modulatory system. Specifically, the functional connectivity of the descending pain modulatory system dependence upon mu-opioid receptor A118G polymorphisms was investigated. This study found that patient groups with different alleles for the A118G polymorphisms exhibited varying functional connectivity between the anterior cingulate cortex and periaqueductal gray (34).

Although magnetic resonance imaging provides information regarding structural and metabolic changes that provide insight into pain perception of the CNS, magnetic resonance imaging cannot image opioid function in cells *in vivo* at the molecular level. Such important opioid function information can be obtained through positron emission tomography and can be used to investigate pain opioid mechanisms (35).

## Inflammation Pain Biomarkers

### Peripheral Neuroinflammation Mechanisms

While the development of neuropathic pain has long been ascribed to the known contributors of central sensitization (action potential kinetics, phenotypic transformation, receptor density zone reorganization and long-term potentiation), the role of neuroinflammation regarding the initiation and maintenance of neuropathic pain has evolved tremendously over the last decade. Pro-inflammatory cytokines have been implicated in the generation of neuropathic pain states at both peripheral and central nervous system sites (36, 37). Neuroinflammation of the peripheral nervous system is triggered by inciting damage to the peripheral nerves, either by trauma, metabolic disturbances (i.e., diabetes), viral infection (i.e., HIV) or surgical lesions leading to sprouting of new pain-sensitive fibers (such as A-β fibers), excessive neuronal firing, and hypersensitization of primary afferent peripheral neurons. During a peripheral nerve injury, local cytokines recruits macrophages which secrete components of the complement cascade, coagulation factors, proteases, hydrolases, interferons, and other cytokines that ultimately facilitate degradation and phagocytosis of the pathogen and injured tissue. Peripheral neuroinflammatory mechanisms affect the damaged neuron and neighboring afferent neurons sharing the same innervation territory (38–45).

### Central Neuroinflammation Mechanisms

Peripheral nerve injury causes neuroinflammation in the spinal cord (41, 46–48). The neuroinflammation is triggered by hyperactivity of the injured primary afferent peripheral sensory neuron which increases neurotransmitters and neuromodulators, causing hyperactivity of postsynaptic nociceptive neuronal hyperactivity as well as the release of several inflammatory activators. A result of this lumbar spinal inflammation process is disruption of the blood-spinal cord barrier leading to increased permeability, which then leads to infiltration of immune cells such as T lymphocytes, macrophages, mast cells, and neutrophils from the periphery into the spinal cord and dorsal root ganglion (46). These mechanisms contribute to further release of inflammatory mediators which contribute to alterations in post-synaptic receptors. This neurotransmitter increase leads to hyperactivity of post-synaptic nociceptive neurons in the spinal cord and altered signaling up to the thalamus and cortex that may contribute to central sensitization and pain hypersensitivity (41, 45, 49). Nerve injury typically involves neuro-immune interaction involving glia (50–52). Glia are known to provide functional microenvironment modulating neuronal signal transduction, synaptic pruning, and neuroplasticity that contributes to central sensitization.

### Inflammation Pain Biomarkers

#### Cytokines as Pain Biomarkers

Concentrations of CSF proinflammatory cytokines are increased in multiple neuropathic pain states (53). Most studies of pain syndromes have found elevations of proinflammatory and anti-inflammatory cytokines in painful conditions compared with healthy controls; furthermore, frequently higher levels of proinflammatory markers are associated with greater pain (54). Other markers such as soluble intercellular adhesion molecule-1 (sICAM-1), for example, have also been demonstrated to correlate with pain. sICAM-1 measured in serum correlates with patients' self-reported pain levels in various pain conditions (back pain, polyneuropathy, post-herpetic neuralgia, orofacial pain, mixed pain, and musculoskeletal pain), distinguishing these patients from those with no or mild pain (55).

#### Cytokines in Peripheral Neuropathy

Cytokines have also been demonstrated to be potent mediators of pain in peripheral neuropathy. In one peripheral neuropathy study, gene expression of pro- and anti-inflammatory cytokines was shown to be increased in patients compared to controls (56). Another study found neuropathic pain group was found to have higher serum levels of several markers including C-Reactive Protein (CRP) and Tumor Necrosis Factor (TNF)-α compared with two control groups. Furthermore, patients with painful neuropathy had higher sICAM-1 and CRP levels when compared to painless neuropathy (57). A meta-analysis comprehensively assessed the relationship between serum TNF-α levels and diabetic peripheral neuropathy in patients with type 2 diabetes, demonstrating increased serum TNF-α levels in patients with diabetic neuropathy compared to type 2 diabetic patients without neuropathy and compared with controls (58). Il-17 is significantly upregulated in rat models of neuropathic pain, and mRNA expression levels of IL-1β and IL-6 are significantly enhanced in the spinal dorsal horn compared with controls (59). Moreover, functional recovery from neuropathic pain following a peripheral nerve injury relies on downregulation of IL-1 β and TNF- α responses (60).

#### Substance P and Neuropeptides Pain Biomarkers

Another key pro-inflammatory neuropeptide, Substance P, is known to initiate biological inflammatory effects (61). In painful trigeminal neuralgia, levels of Substance P and other neuropeptides (CGRP and VIP) in the cerebrospinal fluid and blood of patients were found to have higher levels than that of controls; furthermore, blood levels of these markers correlated with those of the CSF (29). Another study investigating non-neuropathic experimental pain found altered substance P levels and dynamics when comparing older and younger adults (27).

#### Imaging Neuroinflammation Biomarkers

Compromised BBB can be identified with gadolinium-enhanced MRI as is seen in the setting of white matter lesions in multiple sclerosis. CNS-infiltration of circulating immune cells, such as monocyte infiltration into brain parenchyma, can be tracked with iron oxide nanoparticles and MRI. Pathological consequences of neuroinflammation such as apoptosis can be imaged with PET [99mTc] Annexin V or with iron accumulation with using MRI T2^*^ relaxometry. These imaging techniques can be used to image human neuroinflammation which have potential to impact patient care in the foreseeable future (62, 63). Integrated positron emission tomography-magnetic resonance imaging and the radioligand 11C-PBR28 for the translocator protein (TSPO) can be used to image regional brain volumes with glial activation. Given the putative role of activated glia in the establishment and or maintenance of persistent pain, pathophysiology, and management of a variety of persistent pain conditions the results from this technique are important to consider when considering imaging techniques for measuring CNS inflammatory effects of pain (64).

## Endocannabinoid Pain Biomarkers

### Endocannabinoid Mechanisms

There are three classifications of cannabinoids: phytocannabinoids (plant-derived), endocannabinoids (in human or animal tissues), and synthetic cannabinoids. Similar to the opioid system, versions of the endocannabinoid (ECB) system have been found in the vast majority of species with a nervous system (65). In particular, the ECB ligands 2-AG and AEA have been found throughout the animal kingdom (66). The ECB system regulates physiology across most organ systems and operates independently and interacts with the inflammatory system, the opiate system, the Vaniloid (TRP) system, and with nuclear transcription factors (67–72). The ECB system works as a part of a negative feedback loop that regulates neurotransmitter and neuropeptide release in the nervous system. Endocannabinoid ligands are generated on-demand in response to high levels of activity and produce short-term inhibitory effects *via* their actions as retrograde transmitters at presynaptic inhibitory G protein-coupled receptors (2).

The two most prevalent endocannabinoid ligands that bind endocannabinoid receptors are anandamide (AEA) and 2-arachidonoylglycerol (2-AG) (67). The 2-AG basal level is ~1,000 times greater than AEA in the brain (73). The enzyme acylphosphatidylethanolamine-phospholipase D is involved in the formation of AEA, and the enzyme diacylglycerol lipase is involved in 2-AG formation (74). Once synthesized and released, endocannabinoids are removed from the extracellular space through an endocannabinoid membrane transporter, subsequently AEA is hydrolyzed by the enzyme fatty acid amide hydrolase (FAAH), and 2-AG is degraded by cytosolic monoacylglycerol lipase (MAGL).

There are two G protein-coupled ECB receptors (CBR1 and CBR2). CBR1 receptors are highly expressed on presynaptic neurons in the brain, spinal cord, and dorsal root ganglion. CBR2 receptors are primarily expressed in immune cells (including myeloid, macrophage, lymphoid, and mast cells) (75). AEA maintains basal endocannabinoid tone and has a high selectivity for the CBR1 receptor over the peripheral CBR2 receptor.

The CBR1 receptor is the most abundant G protein-coupled receptor in the brain and one of the most abundant in both the peripheral and central nervous system. CBR1 are expressed primarily on presynaptic peripheral and central nerve terminals. CBR1 is the central receptor responsible for the behavioral and psychotropic effects of the “high” caused by THC (65, 72). The CBR1 receptor is also present in multiple immune cells, making it important when considering anti-inflammatory properties of endocannabinoids (68, 75).

The CBR2 receptor is largely present on peripheral immune cells and participates in regulation of the immune system. The principal endogenous ligand for the CBR2 receptor is 2-AG. In the brain, stimulation of CBR2 receptors does not produce cannabis-like effects (72, 74). The anti-inflammatory effects of exogenous cannabinoids are mediated by the endocannabinoid system, likely through CB2Rs in the periphery that have immunomodulatory functions (76).

In neural and non-neural systems, in response to tissue injury or excessive nociception, the ECB system generally suppresses inflammation, suppresses sensitization, and suppresses pain (68, 71, 72, 77); however, ECB activity on the nociception system can be complicated, with ECB antinociceptive or pronociceptive depending on the site of expression and the underlying physiological brain state (2).

### Endocannabinoid Pain Biomarkers

ECB biomarkers have been used to monitor neuropathic pain. Increases in circulating AEA concentrations occur in patients with neuropathic pain complex regional pain syndrome (CRPS) when compared to controls without pain (78). Increases in circulating 2-AG concentrations have also been reported in neuromyelitis optica (79). Circulating concentrations of AEA and 2-AG have been shown to correlate with the numbers of daily severe headaches (80). ECB biomarkers also are present for non-neuropathic pain conditions: bladder pain (81), fibromyalgia (78, 82), cold pain sensitivity (83), osteoarthritis (84), knee pain (85), and back pain (86). Clinical Cannabinoid Deficiency Syndrome has been linked to migraines, neuromuscular pain, and gastrointestinal disorders (87).

### Endocannabinoid Interactions With Inflammation and Opioids

When considering ECB pain biomarkers, it is important to consider that the ECB system interacts significantly with inflammation mechanisms as well as opioid mechanisms. The ECB system regulates inflammation at multiple levels and generally inhibits inflammation. In preclinical and laboratory investigation, it has been shown that the ECB system inhibits pro- inflammatory cytokines while increasing production of anti-inflammatory cytokines. The ECB system also inhibits immune cell activation, immune cell proliferation and migration, and can increase immune cell apoptosis *via* multiple mechanisms (23, 67, 88–93).

CBR1 receptors are 10 times more concentrated than mu-opioid receptors in the brain, and cannabinoid receptors co-localize with opioid receptors in many regions involved in pain circuitry including the dorsal horn of the spinal cord and in the supra-spinal periaqueductal gray and rostro-ventral medulla (67, 72, 94). ECB and opioid pain biomarkers potentially can be identified *via* endogenous opioid function which can be assumed when comparing pain sensitivity in the presence of opioid blocking treatment, such as naloxone vs. placebo. Higher endogenous opioid function is associated with decreased benefit from opioid treatments such as morphine (95). Exercise induced analgesia involves both opioid and endocannabinoid mechanisms (96). Exercise induced increases of endocannabinoid ligands such as AEA are blocked by naltrexone. This indicates that opioids are involved in the increase of endocannabinoid ligands following exercise (97). Intrathecal morphine decreases circulating levels of endocannabinoids demonstrating how the opioid and endocannabinoid systems are linked (98). Lower endogenous opioid function is associated with greater analgesia from pain treatment with opiates. One study demonstrated that low endocannabinoid activity is also associated with greater analgesia from pain treatment with opiates (99).

Pathogenic alterations in the distribution of microbial species within the gut (gut dysbiosis) is associated with neuropathic pain in a variety of clinical conditions. One study found that reductions in the diversity and increases in the ratios of two microbial species (ratios of Blautia and Clostridium to Lachnospira) may contribute to HIV-associated neuropathic pain (100). This may be particularly relevant in the context of the endocannabinoid system, as the endocannabinoid system regulates homeostasis of multiple organ systems, including the gut. Because dysregulation of the gut-brain axis can result in chronic inflammation and neuroinflammation, endocannabinoids have anti-oxidant, and anti-inflammatory properties relevant to modulation of inflammation that occurs along the gut-brain axis (101, 102).

## Non-Imaging Pain Biomarkers

### Genetic Biomarkers

In a systematic review and meta-analysis of 29 studies on potential genetic variants associated with neuropathic pain identified (103), 28 genes were significantly associated with neuropathic pain, many involved in neurotransmission, immune response, and metabolism. Genetic variants in *HLA* genes*, COMT, OPRM1, TNFA, IL6*, and *GCH1*, were found to have an association with neuropathic pain in more than one study. In the meta-analysis, polymorphisms in HLA-DRB1^*^13, HLA-DRB1^*^04, HLA-DQB1^*^03, HLA-A^*^33, and HLA-B^*^44 were associated with significantly increased risk of developing neuropathic pain, whereas HLA-A^*^02 reduced risk of neuropathic pain.

To detect genetic associations, particularly those of small effect size, a study must be sufficiently statistically powered to detect those differences. Most genetic studies of neuropathic pain have typically analyzed cohorts with <1,000 cases, which has resulted in only suggestive associations (103, 104). One reason that genetic studies in neuropathic pain lack sufficient sample sizes is the costs associated with studying these large cohorts (105).

### Micro-RNA

#### Micro-RNA Mechanisms

MicroRNA are small non-coding RNA molecules that contain about 22 nucleotides and are found in plants, animals, and some viruses (106). Identification of the first microRNA occurred in 1993, and currently more than 2,000 human microRNAs have been recognized (107). MicroRNA function in RNA silencing and post-transcriptional gene expression regulation. Base-pairing occurs between microRNA and complementary sequences of mRNA molecules leading to silencing of mRNA by (i) cleavage of the mRNA, (ii) destabilization of the mRNA by shortening the poly(A) tail, and (iii) inefficient translation of mRNA into proteins by ribosomes. Each microRNA species regulates multiple genes creating a complex regulatory network (108, 109).

MicroRNA-mRNA interactions allow for modification of gene expression by controlling translation in response to signaling events. Disease states or perturbations in cellular homeostasis can lead to aberrant microRNA expression (110). Numerous studies suggest the involvement of microRNAs in key biological processes including development and cellular homeostasis, and their altered expression is associated with various pathological conditions including cancer, immune disease, inflammatory disease as well as pain mechanisms (106, 111, 112).

#### Micro-RNA Are Stable in Blood Circulation

It is well-established that microRNAs are present in the serum and plasma of humans and stable (as part of RNase resistant molecular complexes or within vesicles or exosomes) such that retrospective studies can be performed using banked samples (106, 110, 113). Horizontal transfer of circulating microRNAs between cells is a novel mode of intercellular communication (109).

#### Micro-RNA-Based Biomarkers of Chronic Pain

The discovery of stable microRNAs in circulation has generated enormous interest in exploring their utility as potential non-invasive biomarkers (110, 114). The induction and chronification of pain are associated with many expressional changes in pain-related proteins regulated by microRNA. Thus, microRNAs are useful as diagnostic and prognostic biomarkers in pain medicine. MicroRNAs have been found to be involved in the onset and progression of several human chronic pain conditions by means of gene repression (107, 114–116). MicroRNA signatures specific to different pain conditions, and their reversal on treatment can be beneficial in patient stratification, prognosis and in bridging pre-clinical, and clinical results (113).

Dysregulations in microRNAs have been reported in several pain disorders in humans in both affected tissues and the circulation (107, 117): Neuropathic pain (117, 118), peripheral neuropathy (119, 120), complex regional pain syndrome (109, 117, 118), cystitis-induced chronic pain (118), osteoarthritis (121), irritable bowel disorder (117, 118), fibromyalgia (117, 118), and migraine (107, 111, 122).

#### Micro-RNA Biomarkers of Chronic Pain Treatment

Presence of circulating microRNAs within exosomes opens up novel avenues for targeting treatments for chronic pain conditions. Such approaches can provide insights on the molecular underpinnings regarding therapeutic targets, treatment doses, and patient eligibility for different treatments (113, 118, 123).

Selectively inhibiting or supplementing a microRNA contributing to pathogenesis is being pursued as a therapeutic strategy for a variety of disorders. Studies from rodent pain models and from patients have now implicated a role for microRNAs in mediating various aspects of pain processing. These non-coding RNAs can provide mechanistic insights into the pathways modulated and could serve as therapeutic targets (110). Drug treatments alter microRNAs in humans and in various animal models. Thus, microRNAs can be predictive biomarkers for therapeutic intervention as well as prognostic markers for treatment response (106).

#### Micro-RNA Biomarkers of Painful Peripheral Neuropathies

MicroRNA modulated inflammation has a major role in the induction and maintenance of neuropathic pain. Inflammation-regulating microRNA profiles in patients with peripheral neuropathies have been characterized. In patients with polyneuropathies of different etiologies the expression of miR-21-5p, miR-146a, and miR-155 were upregulated. In painful neuropathies, tissues from skin biopsies from the lower leg, where neuropathic changes are most common, had reduced miR-146a and miR-155 expression compared to the thigh; furthermore, peripheral neuropathies are associated with aberrant microRNA expression in the sural nerve and in the skin (119). In sural nerve biopsies of patients with peripheral neuropathies miR-132-3p expression was more than doubled in white blood cells of neuropathy patients compared to healthy controls as well as in painful compared with non-painful neuropathy (120). MiRNA's (miR-155) have been found to be upregulated in polyneuropathies, and miR-21 is increased in painful neuropathies.

### Stress

#### Allostatic Load

Allostatic load indices, an index that measures the effects on the body by chronic stress, have been demonstrated to predict morbidity and mortality. Allostatic load has been studied in pain with allostatic load model covariates including age, sex, education, smoking status, alcohol consumption, activity level, depression, and common comorbid health conditions. Allostatic load pain models have shown a positive relationship between pain severity and allostatic load (124).

#### Cortisol and Dehydroepiandrosterone

Cortisol is a proposed stress-related pain biomarker (125). DHEA and DHEAS are neurosteroids that modulate inhibitory GABA receptors and excitatory NMDA receptors, producing complex neuronal effects (126). In animal studies, DHEA and DHEAS levels have been proposed as a biomarker for pain (127, 128). In multivariable regression analysis, gender, age, and pain perception in the shoulder and upper limbs were significantly related to serum DHEAS (129). In another study plasma DHEAS levels were lower compared with persons with chronic neck pain compared with controls with no pain (130). One study found that the odds of having depressive symptoms increased with higher cortisol/DHEA-S ratios among people living with HIV on treatment, suggesting altered neuroactive steroid metabolism may contribute to the pathophysiological mechanisms of depression in people living with HIV (131). A study of male war veterans found that reductions in DHEA levels were associated with muscle soreness and were positively associated with chest pain (132). Self-reported back pain measures in female war veterans were inversely correlated with DHEA and DHEA-S (126); those reporting moderate to severe low back pain demonstrated significantly lower DHEA-S levels compared to those with no or mild lower back pain.

#### Allopregnanolone

Allopregnanolone is a neuroactive steroid derived from progesterone that is synthesized within the nervous tissue. Allopregnanolone interacts with GABA-A receptors making it important in neuroprotection particularly in cases of ischemia and peripheral neuropathy. Plasma allopregnanolone immunoreactivity has been associated with decreased pain sensitivity in humans which may be mediated by hypothalamic-pituitary-axis function (133). Allopregnanolone levels have also been inversely associated with low back pain and chest pain (132). In addition, allopregnanolone levels have been inversely associated with muscle soreness, chest pain, and aggregate total pain among war veterans (134).

### Saliva

Biomarkers in saliva may be useful as they are easily measurable without requiring a needlestick or invasive methods (135). Salivary biomarkers, such as salivary cortisol, salivary α-amylase, secretory IgA (sIgA), testosterone, glutamate, or tumor necrosis factor receptor type II (TNF-RII) has been proposed as possible pain biomarkers (136–138). In particular, sIgA and TNF-RII as useful salivary markers of pain given their high intra-individual reproducibility (139, 140).

### Other

#### QST and Skin Biopsy and Peripheral Nerve Imaging

Quantitate sensory testing and skin punch biopsy results are potential peripheral neuropathic pain biomarkers, in particular for diabetic peripheral neuropathy (141, 142). Markers for peripheral nerve fiber degeneration and regeneration, microvasculature characteristics, and peripheral angiogenesis have been investigated as biomarkers for diabetic peripheral neuropathic pain (142). A review of MRI imaging of the sciatic nerve and its branches provides convincing evidence that diabetic peripheral neuropathy is associated with increase nerve cross sectional area, T2-weighted hyperintense and hypointense lesions, evidence of nerve edema, decreased fractional anisotropy and increased apparent diffusion coefficient. These nerve abnormalities are potential markers of pain in diabetic neuropathy (143).

#### Physiological Markers

Skin conductance responses and alterations in electrocardiograms have been used to predict pain level ratings with high sensitivity and moderate specificity (144). Pupillary dilatation in response to noxious stimuli is thought to be related to locus coeruleus responses to nociceptive stimuli (145).

#### Fatty Acids and Linoleic Acid Derivative

Ornithine levels have also been found to be elevated in patients with persistent muscle pain (146). Significant correlations have also been seen for plasma concentrations of the linoleic acid derivatives 9- and 13-hydroxy-octadecadienoic acid among patients with neck pain (147).

#### Neurotrophic and Neurotransmitter Pain Biomarkers

Neurotrophic factors (BDNF, NGF, NT3, TrkA) and erythropoietin with the erythropoietin receptor are up-regulated in patients with peripheral neuropathy (56). Neurotransmitters in serum have been proposed as pain biomarkers, including neuropeptide Y and BDNF (148–150) as well as Dopamine (151). The catecholamine product metanephrine has also been proposed as a pain biomarker (152).

## Brain Imaging Pain Biomarkers

### Pain Brain Circuits

**Figure 1:**
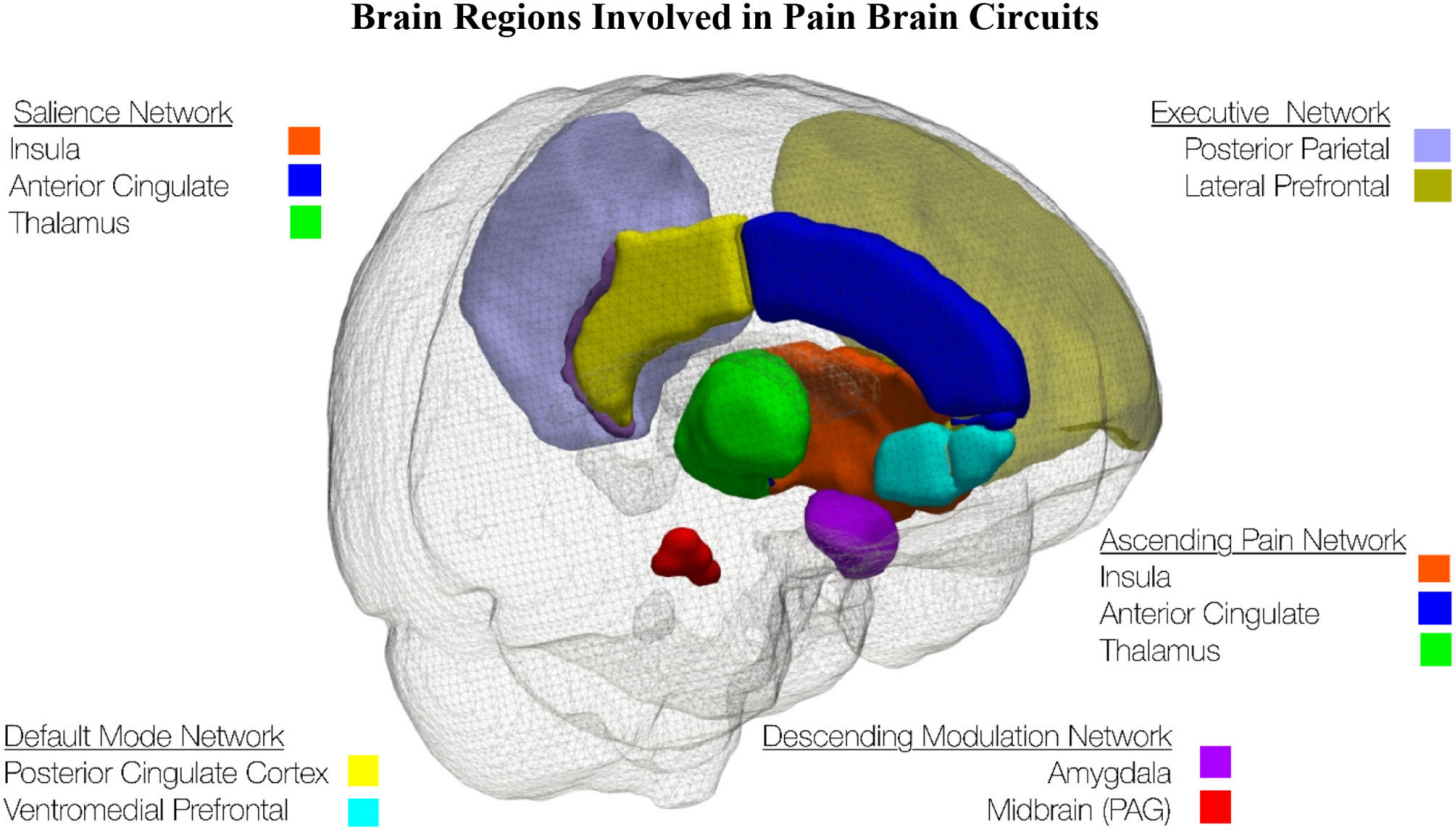
This schematic of the left side of the brain shows brain regions involved in pain brain circuits. The brain regions were extracted from the Hammers Maximum Probability Atlas (153).

In the past decade, the focus of brain imaging investigation of pain mechanisms has shifted from investigating individual regions of the brain to investigating brain circuits, see Figure 1. One of the most important brain circuits is the default mode network which is associated with daydreaming (154, 155). The executive network is the brain circuit used when not daydreaming but instead attending to the outside world (156). The ascending pain network includes the anatomical pathway that conveys the nociceptive input from the peripheral nervous system to the spinal cord and the brain (157). The descending modulation network involves brain regions that connect to the brainstem and then down to the dorsal horn and increase or decrease ascending pain signals depending on the behavioral state of the individual (158). The salience network is a large-scale brain network of the human brain that is primarily composed of the anterior insula and dorsal anterior cingulate cortex. It is involved in detecting and filtering salient stimuli, as well as in recruiting relevant functional networks (157, 159).

### Brain Circuit Changes in Chronic Pain

#### Chronic Pain Reduced Gray Matter Volumes

One of the most established chronic pain brain imaging biomarkers is reduced regional gray matter volumes in brain regions related to pain processing (160–164). One of the early reports of reduced brain gray matter volumes in chronic pain was reported in chronic back pain (160). Despite that brain atrophy has been identified as a potential cause of chronic pain in neurologic dementia disease (165), it has been demonstrated that chronic pain causes brain atrophy (164). Subsequent studies identified characteristic patterns of gray matter atrophy in different chronic pain conditions (161–163).

#### Presence of Chronic Pain

It has been shown that in multiple chronic pain conditions that there is increased connectivity between the salience network and the default mode network and decreased activity in the default mode network (166–171). This exciting biomarker for chronic pain indicates that the chronic pain experience is disrupting the normal daydreaming default mode network with an abnormal interaction with the salience network which monitors stressful events like the presence of painful stimulation.

#### Pain Rumination

Pain rumination occurs when people negatively dwell on their pain experience. Interestingly, this negative cognitive dwelling on the pain experience appears as increased activity in the default mode network (172). This increase in default mode activity is distinct from the decreased activity observed in the default mode network when chronic pain increases cross-talk between the salience network and the default mode network (166).

#### Pain Mind Wandering

It is plausible to envision that healthy mind wandering away from thinking about the pain experience is the opposite of pain rumination (172, 173). The dynamic pain connectome is a model that helps to understand the pain experience that include the salience network, default mode network, ascending pain system, and descending pain modulation pathway (157, 159). The dynamic pain connectome model was derived from brain imaging data in healthy subjects who had mind-wandering away from a painful stimulus. This work found that most brain regions were activated by noxious stimuli whether the mind wandered away from the stimulus or not. The responses of the salience and default mode networks and connectivity with antinociceptive areas showed mind wandering brain activity that included a clear distinction between trials in which subjects attended to pain vs. mind-wandered from pain (159, 173).

#### Transition to Chronic Pain

Increased functional connectivity between the medial prefrontal cortex–nucleus accumbens at the beginning of back pain predicts that patients will go on to develop chronic back pain; while patients with decreased connectivity in this circuit went on to recover from back pain (174, 175). Structural brain imaging in subacute back pain patients was followed longitudinally for 3 years as they either recovered from or transitioned to chronic pain. Furthermore, these results indicate that persistence of chronic pain is predetermined by corticolimbic neuroanatomical factors (176).

#### Placebo Mechanisms

There is substantial overlap between the circuits involved in human placebo analgesia and those that mediate multiple forms of context-based modulation of pain behavior in rodents, including forebrain-brainstem pathways and opioid and cannabinoid systems in particular. This suggests that placebo effects are a set of adaptive mechanisms that shape nociceptive signaling (177, 178). Subcortical limbic volume asymmetry, sensorimotor cortical thickness, and functional coupling of prefrontal regions, anterior cingulate, and periaqueductal gray are predictive of placebo response (179). One study found that placebo and nocebo effects are generated through differential engagement of the periaqueductal gray-rostral ventromedial medulla pathway, which likely influences pain experience by modulating activity at the dorsal horn level (180).

### Pain States and Traits and Resilience

Individuals have a set point around which different biological attributes can fluctuate transiently into different states. However, if one remains in a different state other than their set point for a considerable period (e.g., induced by a disease), this different state is considered a new set point. In pain research it is important to consider trait and state pains to gain an understanding of not only an individual's current pain state but also more broadly to their trait pain, which may be more reflective of their general condition (181). Resilience is a trait that is highly associated with chronic pain–related health outcomes. The neural correlates of both pain and trait resilience are critical to understand the brain–behavior relationship in chronic pain; yet, neural correlates of resilience in chronic pain states are unknown (182). Therefore, regional BOLD (Blood Oxygen Level Dependent) variability and circuit connectivity have potential to provide predictive power for pain resilience or vulnerability to chronic pain and treatment efficacy (182, 183).

### Imaging Biomarkers for Diabetic Peripheral Neuropathic Pain

Two reviews on mechanisms and imaging biomarkers for diabetic neuropathic pain review that diabetic peripheral neuropathy and associated pain have structural and functional central nervous system changes in the spinal cord, subcortex, and cortex (184, 185). Diabetic peripheral neuropathy has been associated with changes in the thalamus. A decreased thalamic NAA/creatinine ratio is suggestive of thalamic neuronal dysfunction (186, 187), and thalamic microvascular perfusion changes have also been observed (188). Smaller spinal cord cross-sectional area has been observed in those with diabetic peripheral neuropathy (189, 190). In fact, in diabetic peripheral neuropathy, diffusion tensor imaging techniques found posterior column damage in the cervical spinal cord (191). Diabetic neuropathic pain is related to decreased NAA in the thalamus (187), increased thalamic vascularity (188), and spinal cord posterior column damage (191).

Diabetic neuropathic pain is associated with increased regional brain gray matter volume loss localized to brain regions involved in somatosensory perception (192); furthermore, in diabetic neuropathic pain, increased total gray matter atrophy is associated with impaired ability to walk (193). Diabetic neuropathic pain has been shown to be related to aberrant default mode functional connectivity (171), decreased functional connectivity between the thalamus and cortex (194), and decreased functional connectivity in attention networks (195). Altered fMRI activation responses to experimental heat pain in limbic and striatal brain circuits are related to the duration of diabetic neuropathic pain (196). Diabetic neuropathic pain is related to a double dissociation such that neuropathic pain intensity is more associated with thalamus-insular cortex functional connectivity and nerve deficits are more related to thalamus-somatosensory cortex functional connectivity (197). Diabetic neuropathic pain is also associated with decreased functional connectivity between the thalamus and amygdala (198), decreased gray matter volumes and decreased white matter connectivity in pain processing and pain modulation brain regions (199), decreased somatosensory cortical thickness related to cortical function dysfunction (200), increased activity in the anterior cingulate cortex (201), as well as ventrolateral periaqueductal gray functional connectivity is altered and correlates with magnitude of spontaneous pain and allodynic pain (202).

### Imaging Biomarkers for HIV Peripheral Neuropathic Pain

Structural brain imaging has revealed changes to the brain associated with HIV peripheral neuropathy. Total cortical volume is smaller with HIV distal neuropathic pain (203). In fact, in HIV distal neuropathic pain the posterior cingulate cortex is the cortical region that was found to be smaller (204). In another sample of people living with HIV, subjective symptoms of HIV peripheral neuropathy were associated with smaller precuneus volumes which overlap with the posterior cingulate cortex (205). Smaller brain volumes for HIV distal neuropathic pain are consistent with a general pattern that brain volumes are reduced for a variety of chronic pain conditions (161, 162). Interestingly, the midbrain, thalamus and posterior cingulate cortex volumes are all reduced in HIV distal neuropathic paresthesia (206). It has been suggested that brain atrophy associated with HIV distal neuropathic paresthesia may precede brain atrophy associated with HIV distal neuropathic pain (206).

More recent multi-modal brain imaging research has revealed structural brain changes associated with HIV peripheral neuropathy (207). HIV peripheral neuropathy is correlated with decreased white matter integrity running from the midbrain to the somatosensory cortex. HIV peripheral neuropathy severity is also associated with decreased generalized fractional anisotropy along the tracts of the external capsule in both hemispheres, appearing to lead along the lateral thalamus to sensorimotor cortex. A similar correlation is found in the superior bilateral cingulum. These results indicate ascending deafferentation in HIV peripheral neuropathy extends further downstream from damaged peripheral nerves than seen previously, into the cortex.

HIV-associated distal neuropathic pain is associated with decreased fMRI resting state functional connectivity in the default mode network and increased functional connectivity in the salience network (208). Decreased connectivity between the medial prefrontal cortex and posterior cingulate cortex and stronger connectivity between the ACC and thalamus is associated with HIV distal neuropathic pain.

In the setting of experimental heat pain, significant interaction has been found within the right anterior insula during expectation of experimental pain offset in that a group with HIV distal neuropathic pain compared group without HIV distal neuropathic pain exhibited increased insula activation in the feet (with painful neuropathic pain) compared to the hand (without neuropathic pain) (209). These findings are consistent with abnormal processing of expectation of experimental pain offset or abnormal pain relief mechanisms potentially due to increased negative expectation regarding the experience of chronic endogenous neuropathic pain.

### Imaging Biomarkers for Chemotherapy Peripheral Neuropathic Pain

Anterior cingulate cerebral perfusion and gray matter density correlate with chemotherapy-induced peripheral neuropathy symptoms including pain (210). Patients with chemotherapy-induced peripheral neuropathy symptoms (including pain) demonstrated greater activation during painful stimulation in the precuneus compared to healthy controls and exhibited hypo-activation of the right superior frontal gyrus compared to healthy controls. Painful stimuli delivered chemotherapy-induced peripheral neuropathy symptoms patients evoke differential activation of distinct cortical regions, reflecting a unique pattern of central pain processing compared with healthy controls providing a tool for monitoring cerebral changes during anti-cancer and analgesic treatment (211).

### Small-Fiber Peripheral Neuropathy Brain Changes

A population of mixed small-fiber peripheral neuropathy (metabolic, inflammatory, chemo, idiopathic) was used to investigate how dysfunction of skin nerves led to abnormal recruitment of pain-related brain regions, suggesting that the brain may be affected in SFN. Greater volume reduction in pain-processing regions, particularly the bilateral anterior cingulate cortices was associated with greater depletion of intraepidermal nerve fibers. There was significant reduction in functional connectivity from the anterior cingulate cortex to the insula pain-processing cortex that is linearly correlated with the severity of intraepidermal nerve fiber depletion (212). Similarly, another population of mixed small-fiber peripheral neuropathy (metabolic, inflammatory, chemo, idiopathic) the degree of skin nerve degeneration was associated with the reduction of connectivity between the thalamus and pain-related areas. Despite altered white matter connectivity, there was no change in white matter integrity assessed with fractional anisotropy. These findings indicate that alterations in structural connectivity may serve as a biomarker of maladaptive brain plasticity that contributes to neuropathic pain after peripheral nerve degeneration (213).

### Imaging Biomarkers for Other Peripheral Neuropathies and Pain

A population of Charcot-Marie-Tooth patients had abnormal diffusion tensor imaging findings indicative of significant cerebral white matter abnormalities. Diffusion tensor imaging abnormalities were correlated with clinical disability, suggesting that there is comorbidity of central nervous system damage with peripheral neuropathy in Charcot-Marie-Tooth patients (214). A population of patients with hereditary neuropathy with liability to pressure palsies were compared to a population of normal controls and the fractional anisotropy values of the patients were significantly lower in bilateral frontal, orbitofrontal, and temporal areas of white matter (215). Patient populations of paresthesia-dominant and pain-dominant patient groups were compared and contralesional cortical thickness were correlated with pain severity (216). Acquired and hereditary peripheral neuropathies are associated with increased functional connectivity of the left precuneus/posterior cingulate cortex in the default mode network. This increased connectivity in the default mode network is correlated with duration of peripheral neuropathy and severity of clinical total neuropathy score (217).

## Composite Pain Biomarkers

As discussed in the introduction, if used in combination, biomarkers related to pain mechanisms offer the possibility to develop objective pain-related indicators that may help diagnosis, treatment, and understanding of pain pathophysiology (8, 10). One possible application of such an approach might be to determine if a patient who is not communicative is experiencing pain. Another example may be to help guide selection of treatment for neuropathy, such as whether transcranial magnetic stimulation may alter network activity among those with neuropathy.

Modeling pain brain mechanisms can be achieved using multi-modal brain imaging including functional magnetic resonance imaging, structural magnetic resonance imaging, diffusion tensor magnetic resonance imaging, electroencephalography, EMG, and PET (10, 13, 14, 218). As we have reviewed here, in addition to using imaging biomarkers, composite pain biomarkers can be investigated using a multitude of non-imaging biomarkers.

Multiple analytic approaches have been used to investigate composite pain biomarkers: (i) composite algorithms have been investigated (219), (ii) unsupervised and supervised multivariate analyses have been used to distinguish pain groups and non-pain groups (220), (iii) supervised pattern recognition have been used to cluster diagnostic groups for different pain conditions (221), (iv) mechanism-based pharmacokinetic-pharmacodynamic modeling has been used to identify biomarkers that help diagnose pain and predict pain treatment (16), (v) principal component analysis has been applied to biochemical markers to create distinct pain profiles (222), (vi) patterns of inflammatory blood cytokines and chemokines have been used to differentiate pain and non-pain groups (223), (vii) multivariable data analysis using simultaneous analysis of 92 inflammation-related proteins with pain intensity and pain thresholds were used to identify protein patterns which distinguish pain and non-pain groups (223), (viii) metabolomics have been applied to chronic pain (224).

## Discussion

### Methods to Find Composite Pain Biomarkers

As detailed above, chronic pain and neuropathic pain impact multiple organ systems. Advancing the value of pain biomarkers depends on (1) selection of measurements and metrics that are the most mechanistically valid and informative, and (2) combining the selected measurements such that they mechanistically and statistically maximize accurate classification. Advancement of measurement accuracy is vital and the subsequent steps of the approach are entirely contingent upon the success of this step. This literature for the domains discussed in this manuscript is too voluminous for a single review. In the above reviewed literature, we attempted principally to focus on which biological systems and which biomarkers should be the focus of measurement. For effective application of measurements of these domains it is important to discuss approaches for measurement selection.

**Figure 2:**
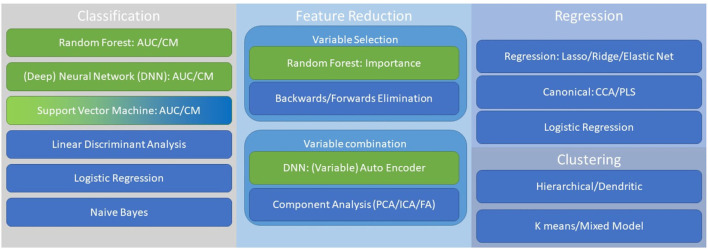
This is a non-comprehensive guide to important approaches when considering multimodal biomarkers. Key approaches include, classification, feature reduction, regression, and clustering. Linear (blue) and non-linear (green) approaches are highlighted, although ranking order and other transformations can be adapted across methodology.

In Figure 2, we provide a significantly abbreviated schematic of key available statistical approaches to handling multimodal datasets in building composite biomarkers. We have highlighted four general areas of statistics/machine learning: (1) feature reduction (225), (2) classification (226, 227), (3) regression (228), and (4) clustering (229). Feature reduction can occur during or prior to classification, regression, or clustering. Feature reduction primarily focuses on two primary approaches: (1) integration of measurements toward creation of a composite variable to simplify and enhance model performance, and (2) effective feature reduction through variable selection to use optimal variables. Thus, feature reduction can represent the effective combining of strong measurements to a meaningful and robust latent variable or elimination of unnecessary, or statistically weak, measurements. Some methods, such as random forest, has built in feature reduction (230). Classification methods are often utilized to build toward categorical variables, however methods like neural networks are also designed for predicting continuous variables (231). Regression models are often used for the prediction of continuous measures or in the case of canonical approaches this can be with multiple dependent variables predicted simultaneously (232). Finally, in the case where there is no existent or optimal category or variable that the biomarkers seek to predict unsupervised approaches can be useful. With all these approaches variables can either be approached as linear or non-linear, although transformations and feature reduction approaches can mitigate these differences. It is important, regardless of approach, to understand the biological mechanisms being modeled by defining a model that best reflects the underlying systems to optimize prediction.

Two key methods for statistical reduction of variables are (1) selecting top ranking variables and (2) creation of composite variables by factor or component-based analysis. Random Forest, as depicted in Figure 2, can be utilized to determine importance scores by evaluating the hierarchical functionality of a given variable as a bifurcator for optimizing classification (226). Random Forest is not alone in its utility to provide variable importance ranking but provides a nice mechanism for this analysis. The statistical creation of composite variables can be done through principal component analysis (or independent component analysis among other methods) such that novel values are calculated for a set of variables that account for large swaths of variance with a single value vector (233, 234). This can substantially increase the efficiency of a model and serve to highlight a robust latent feature.

#### Summary of Possible Pain Biomarkers

A summary of pain biomarkers discussed in this review article are provided in Table 1. Non-imaging pain biomarkers include opioid pain biomarkers: Beta-endorphin, B-cell opioid receptors, composite genetic, Mu-opioid receptor A118G polymorphisms, migraine opioid PET, and endogenous opioid function. Inflammatory pain biomarkers include cytokines, sICAM-1, cytokines related to back pain, cytokines related to peripheral neuropathy, substance P, and neuropeptides. Endocannabinoid pain biomarkers include: AEA in CRPS, 2-AG in optic neuromyelitis, AEA and 2-AG in headaches, ECB elements in multiple non-neuropathic pain conditions, ECB elements in endogenous opioid function, and ECB elements in gut-brain interactions. There are pain biomarker genes related to neuropathic pain risk. MICRO-RNA dysregulation pain biomarkers are found in neuropathic pain, peripheral neuropathic pain, CRPS, migraine, and non-neuropathic pain conditions. Stress related pain biomarkers include allostatic load, Cortisol, DHEA(S), and allopregnanolone. Measuring saliva contains potentially particularly accessible pain biomarkers. Other pain biomarkers can be accessed *via* QST, skin conductance, pupil dilation, fatty acid pain biomarkers (ornithine and linoleic acid derivatives), neurotrophic factors, and serum neurotransmitters.

**Table 1:** Summary table for pain biomarkers.

**Type of pain biomarker**	**Pain biomarker**	**Pain disease**	**Section**	**References**
Opioid markers	Beta-Endorphin	Experimental Neuropathic Trigeminal	2	(27–30)
	B-Cell opioid receptors	Neuropathic	2	(31)
	Composite genetic	Heterogeneous	2	(32)
	Mu-Opioid A118G polymorphisms	Dysmenorrhea	2	(34)
	Migraine PET	Migraine	2	(35)
	Endogenous opioid function	Experimental knee	4	(95–99)
Inflammatory markers	Multiple cytokines	Heterogeneous	3	(53, 54)
	sICAM-1 pain intensity	Heterogeneous	3	(55)
	TNF-α	Diabetic PN	3	(56–58)
	Neuropeptides (Substance P, CGRP, VIP)	Experimental Trigeminal Sickle Cell	3	(27, 29, 61)
	Brain imaging neuroinflammation	Multiple inflammatory	3	(62–64)
Endocannabinoid markers	AEA	CRPS	4	(78)
	2-AG	Optic neuromyelitis	4	(79)
	AEA, 2-AG	Headache	4	(80)
	Multiple components ECB	Heterogeneous	4	(80–86)
	Gut inflammation and ECB	HIV	4	(100–102)
Genetic markers	Genetic risk factors	Neuropathic	5	(103–105)
MICRO-RNA markers	MICRO-RNA dysregulation	CRPS	5	(109)
	MICRO-RNA dysregulation	Heterogeneous	5	(117, 118)
	MICRO-RNA dysregulation	Peripheral neuropathy	5	(119, 120)
	MICRO-RNA dysregulation	Osteoarthritis	5	(121)
	MICRO-RNA dysregulation	Migraine	5	(122)
Stress markers	Allostatic load	Heterogeneous	5	(124)
	Cortisol	Systemic sclerosis	5	(125)
	DHEA, DHEAS	Heterogeneous	5	(126, 129, 130, 132)
	Allopregnanolone	Experimental Heterogeneous	5	(133, 134)
Salivary Markers	Cortisol, alpha-amylase, sIgA, testosterone, sTNR-RII, glutamate	Experimental Heterogeneous	5	(136–140)
Other pain markers	QST, skin biopsy	Peripheral neuropathy	5	(141, 142)
	Sciatic nerve MRI	Diabetic peripheral neuropathy	5	(143)
	Skin conductance	Experimental	5	(144)
	Pupil dilation	Experimental	5	(145)
	Ornithine, linoleic acid derivatives	Heterogeneous	5	(146, 147)
	Neurotrophic factors	Peripheral neuropathy	5	(56)
	Serum neurotransmitters	Back pain	5	(148–152)
Pain brain circuit markers	Ascending pain network		6	(157)
	Descending modulation network		6	(158)
	Default mode network (DMN)		6	(154, 155)
	Executive network		6	(156)
	Salience network		6	(157, 159)
	Acute pain machine learning		1	(12–14)
Pain brain circuit modulation markers	Chronic pain cortical atrophy	Heterogeneous	6	(160–164)
	Chronic pain salience network interaction with default mode network	Heterogeneous	6	(166–171)
	Pain rumination increased DMN activity	Temporomandibular	6	(172)
	Pain mind wandering DMN interaction with descending modulation network	Experimental	6	(173)
	Pain chronification increased connectivity between the MPF and NA	Chronic back pain	6	(174–176)
	Placebo and nocebo mechanisms		6	(177–180)
	Pain trait vs. pain states		6	(181)
	Resilience networks		6	(181–183)
Peripheral neuropathy markers	Thalamic changes (NAA, microvascular)	Diabetic	6	(186–188)
	Spinal cord atrophy	Diabetic	6	(189–191)
	decreased cortical gray matter	Diabetic	6	(192, 193, 199, 200)
	Changed brain circuit connectivity	Diabetic	6	(171, 194, 195, 197, 198, 202)
	Changed fMRI activation	Diabetic	6	(196)
	Decreased white matter integrity	Diabetic	6	(199)
	Changed anterior cingulate blood flow	Diabetic	6	(201)
	Decreased cortical gray matter	HIV	6	(203–205)
	Decreased subcortex	HIV	6	(206)
	Decreased white matter integrity	HIV	6	(207)
	Changed brain circuit connectivity	HIV	6	(208)
	Changed fMRI activation	HIV	6	(209)
	Anterior cingulate perfusion and volume	Chemotherapy	6	(210)
	Changed fMRI activation	Chemotherapy	6	(211)
	Decreased cortical gray matter	SFN	6	(212)
	Changed brain circuit connectivity	SFN	6	(212, 213)
	Decreased white matter integrity	Charcot-Marie-Tooth	6	(214)
	Decreased white matter integrity	Hereditary neuropathy with liability to pressure palsies	6	(215)
	Decreased cortical gray matter	Carpal tunnel syndrome	6	(216)
	Changed Brain Circuit Connectivity	Heterogeneous peripheral neuropathy	6	(217)

Brain imaging pain biomarkers for measuring pain can be evaluated using three different MRI brain methods: gray matter structural imaging, white matter diffusion tensor imaging, and functional brain activation. Brain circuits related to pain mechanisms include an ascending brain circuit, a descending pain modulation circuit, the default mode circuit, the executive network brain circuit, and finally the salience network. Pain mechanisms in the brain can be measured *via* modulation in brain circuits: acute pain machine learning measures of chronic pain, pain rumination, pain mind wandering, placebo mechanisms, pain traits and states, and resilience. HIV peripheral neuropathy changes in the brain include reduced total cortical gray matter and reduced posterior cingulate cortex volume in particular, white matter degeneration, altered resting state networks, and aberrant expectation of pain relief.

By focusing on a broad array of mechanisms and biomarkers, we can uncover important mechanistic connections and interactions across systems. Neuropathic pain is a debilitating condition that has primary, and cascading affects across body systems. Assessment and understanding in an appropriately comprehensive approach are challenging due to the vast and diverse literature and the complexity measurement. This review aims to facilitate navigation of this literature and the appropriate selection of biomarkers for future research.

## Author Contributions

All authors have contributed to the writing and scientific direction of this manuscript. All authors contributed to the article and approved the submitted version.

## Conflict of Interest

The authors declare that the research was conducted in the absence of any commercial or financial relationships that could be construed as a potential conflict of interest.

## Publisher's Note

All claims expressed in this article are solely those of the authors and do not necessarily represent those of their affiliated organizations, or those of the publisher, the editors and the reviewers. Any product that may be evaluated in this article, or claim that may be made by its manufacturer, is not guaranteed or endorsed by the publisher.

## Abbreviations

2-AG: 2-arachidonoylglycerol
AEA: anandamide
CBR: G protein-coupled ECB receptor
CSF: cerebral spinal fluid
CRPS: complex regional pain syndrome
CRP: c reactive protein
DMN: default mode network
DHEA: dehydroepiandrosterone
ECB: endocannabinoid
FAAH: fatty acid amide hydrolase
MAGL: cytosolic monoacylglycerol lipase
MPF: medial prefrontal cortex
NA: nucleus accumbens
QST: quantitative sensory testing
SFN: small fiber neuropathy
sICAM-1: soluble intercellular adhesion molecule-1
TNF: tumor necrosis factor
WBC: white blood cell.
